# Effect of herbal irrigants on surface roughness of intraradicular dentin using quantitative method of 3D surface texture analysis

**DOI:** 10.1038/s41598-024-65245-4

**Published:** 2024-07-04

**Authors:** Sabah M. Sobhy, Heba Abdelfatah, Hanaa M. Elgamily, Nesreen Y. Mohammed

**Affiliations:** 1 Endodontic Department, Faculty of Dental Medicine for Girls, Al-Azhar University, Cairo, Egypt; 2 Faculty of Dental Medicine for Girls, Al-Azhar University, Cairo, Egypt; 3 Restorative and Dental Materials Department, Oral and Dental Research Institutes, National Research Centre, 33 El Bohouth St., Dokki, P.O. 12622, Giza, Egypt; 4 Faculty of Dental Medicine for Girls, Al-Azhar University, Cairo, Egypt

**Keywords:** Herbal irrigants, *Moringa oleifera*, Orange oil, Surface roughness, Scanning electron microscopy, Intraradicular dentin, Diseases, Medical research

## Abstract

Replacing the conventional endodontic irrigants with herbal agents could avoid complications associated with using sodium hypochlorite (NaOCl). Endodontic irrigants alter the surface roughness of the dentinal wall surface, which affects sealer mechanical retention. This study aimed to assess the effect of experimental herbal Moringa oleifera and orange peel extract irrigant on intraradicular dentin (IRD) surface roughness using quantitative 3D surface analysis by scanning electron microscopy (SEM) regarding the smear layer assessment. Sixty human root sections were divided into four groups (n = 15): NaOCl combined with 17% ethylenediaminetetraacetic acid (EDTA); negative control (saline); moringa extract (MO); and orange oil (OO). SEM images were assessed quantitatively for surface roughness (Ra) in the coronal, middle, and apical IRD. The data were analysed by Kruskal–Wallis, Friedman, and Dunn’s tests. All groups showed statistically significant differences (P = 0.007). MO exhibited significantly greater Ra values at the coronal, middle, and apical root levels than OO (P = 0.007, 0.009, and 0.046, respectively). There was no significant change in Ra values at various root levels within each group at P = 0.091, 0.819, 0.819, and 0.549 for the EDTA, saline, MO, and OO groups. Considerable (IRD) surface roughness analysis makes Moringa extract a promising herbal endodontic irrigant alternative to the NaOCl plus EDTA regimen.

## Introduction

One of the main difficulties in endodontic treatment is the complete removal of the smear layer. Smear layer production is the outcome of root canal manipulation during endodontic treatment. Such a layer may be responsible for microleakage and contains high organic content, serving as a supply of nutrients for bacteria^1^. Moreover, microleakage remains a clinical problem and a potential cause of failure^2^. As a result, removing the smear layer and microbial biofilm is a crucial part of endodontic therapy. To conduct this task, various chemicals and medicinal substances are utilized^3,4^.

While chelating agents assist in root canal irrigation, their use may lead to structural changes in the dentin walls of the root and changes in surface roughness^5^. The surface roughness of dentin occurs due to alterations in the calcium-to-phosphorus ratio^6^. A slight increase in surface roughness may aid sealer adhesion to the canal wall. An excessive increase in surface roughness may promote the colonization of bacterial biofilm^7–9^. However, micromechanical bonding necessitates the existence of surface irregularities into which endodontic sealer can penetrate, as these irregularities can be quantified by measuring surface roughness^2^. Consequently, eliminating the smear layer may achieve complete root sealing and lessen the chance of bacterial viability and colonization^6,8^. Ethylenediaminetetraacetic acid (EDTA) efficiently eliminates only the inorganic matter in smear layer^10^. Hence, during root canal therapy, the use of a combination of 2.5% NaOCl and 17% EDTA for additional removal of organic components from the smear layer was recommended^11^.

Unfortunately, these chemicals adversely affect the underlying dentin surface in addition to causing biological hazards^12^. Inhalation of hypochlorite can cause irritation, nausea, vomiting, dizziness, breathing distress, headache, and coughing. High concentrations of hypochlorite during preparation may lead to upper respiratory tract and pulmonary edema. Chlorine gas, resulting from chemical reactions between sodium hypochlorite and other substances such as EDTA, can cause upper respiratory tract symptoms, serious hypoxemia, pneumonia, bronchitis, pulmonary edema, and acute respiratory distress syndrome^13,14^. These unfavourable consequences of chemical irrigants prompted researchers to seek natural, safe, and cost-effective alternatives^13–15^. Herbal agents have recently provided various advantages, including long shelf life, minimal biological hazards, low cost, and lack of bacterial resistance^16^.

Moringa oleifera (MO) extract is a diverse herb that is recommended as an endodontic irrigant. Due to its countless benefits, it is considered a medically superior plant. The leaves of MO exhibit antioxidant, anti-tumor, anti-inflammatory, and antimicrobial properties. They contain phytochemicals such as flavonoids, saponins, tannins, and phenolic acids^17^. It was found that moringa extracts, whether alcoholic or aqueous, might improve bonding to intraradicular dentin^18^. In addition, researchers recommended that MO be used to improve dentin hardness and reduce the amount of the intraradicular dentin (IRD) smear layer, as well as an efficient antibacterial irrigant^14,19^.

Orange oil (OO) which belongs to the *Rutaceae* family, is a peel extract that consists of 94% d-limonene, 3% myrcene, and other organic components. D-limonene is regarded as an environmentally friendly and efficient organic cleaning chemical. Vitamin C and essential oils, which have antibacterial, antioxidant, and anti-inflammatory qualities are also found in OO^20^. Previous research exhibited partial smear layer removal of OO compared to other herbal and NaOCl irrigants in removing the smear layer at the apical region^21^. Moreover, recently a study exhibited the moderate ability of OO to dissolve the smear layer through different regions of IRD compared to other experimental irrigants^22^.

Scanning electron microscopy (SEM) is frequently utilized in qualitative analysis^23–25^. However, this approach is unable to calculate material loss quantitatively due to the inability to determine the third dimension (3D)^26^. Commonly used quantitative techniques for characterizing dentin roughness include 2D contact profilometry^27^and atomic force microscopy (AFM)^27,28^. However, only small surface regions can be assessed with AFM, and 2D profile evaluation is possible with contact profilometry^2^.

The capacity to generate precise, quantifiable surface texture analysis has improved owing to new technologies based on scale-sensitive fractal analysis of high-resolution and three-dimensional surface reconstructions^29^. In addition, it has been used to examine tooth structures and employed for various objectives in biology studies of dental structures^30,31^. Hence, it has opened the door to a variety of clinical applications^29,32^.

The current study aimed to assess the effect of experimental herbal Moringa oleifera and orange peel extract irrigant on intraradicular dentin (IRD) surface roughness using quantitative 3D surface analysis by scanning electron microscopy (SEM) regarding the smear layer assessment. The null hypothesis of this research was that there would be no difference in the effect of MO or OO as experimental endodontic irrigants compared to either a positive irrigant using NaOCl with EDTA or a negative irrigant using saline on surface roughness (Ra) across IRD levels. Additionally, MO and OO as herbal endodontic irrigants were expected to exhibit no difference in surface roughness (Ra) at various parts of IRD.

## Methods

### Preparation of experimental herbal endodontic irrigants

#### Aqueous extraction of *Moringa* oleifera (MO) leaves

The fresh leaves of *Moringa oleifera* were supplied by the Egyptian Scientific Society of the Moringa Trees (National Research Centre (NRC); Dokki; Giza; Egypt). The plants were identified by Prof. Aboelfetoh Mohamed Abdella, NRC, Egypt. The aqueous extraction of MO was prepared at the Technology of Horticulture Crops Department, Agriculture Research Institute (National Research Centre (NRC); Dokki, Giza; Egypt). The collected plant leaves of MO were washed under tap water, followed by distilled water, and dried. They were then put into a mixer or grinder. The resultant powder was mixed individually with distilled water in a ratio of 1:1 (w/v) and left overnight to allow the constituents to get dissolved in water, then filtered through a muslin cloth, and a 100% plant extract solution was prepared. The extract obtained was subjected to vacuum filtration followed by shaking. The processed 100% extracts were poured into the Erlenmeyer flasks, plugged with cotton separately, and heated at 50 ºC for 15 min to avoid contamination. The extracts were further diluted to different concentrations, adding distilled sterile water for further use in the experiment^33^_._

#### Oil Orange (OO) fruit peels extraction

Citrus sinensis orange oil (OO) of the Rutaceae plant family was provided by the dairy microbiology laboratory (Food Industries and Nutrition Research Institute, NRC, Egypt). Orange rinds were peeled off carefully with the help of a sharp razor blade. The samples were checked to ensure that none of the white flesh under the rind was included in the sample because white flesh contains little or no limonene. Each rind sample was cut into smaller pieces and 100 g mass was taken. The sample was initially rinsed with distilled water. The improved steam distillation (ISD) for oil orange (OO) fruit peel extraction used in this study was similar to the method explained by Mercy Nisha et al^34^. The water-washed orange peels were pre-heated in a vessel for about 30 min at a temperature of 50 °C. A little amount of distilled water that is enough to immerse the orange peels is added. The pre-heated sample was then loaded into the round-bottomed flask and two-thirds of it was filled with distilled water including the water that retained after preheating the cut orange peels. The condenser water was turned on and heated up to 100 °C for two hours. Water loss occurred due to continuous heating. If the water level got too low, the sugar present in oranges would caramelize and burn. To avoid this two-thirds of the round-bottomed flask was filled with water. The distillation was then run, for about 120 min, at varied temperature ranges (10 °C, 20 °C and 30 °C) of the heating mantle. The resultant liquid was collected and the presence of the orange oil in the condensate was confirmed by the cloudy appearance on the top of the distillate. Finally, OO was separated from water by adding 15 ml of chloroform in a separating funnel in the presence of sodium chloride to minimize emulsions during this final extraction by making the organic layer less soluble in the aqueous layer. After the chloroform had evaporated completely in water bath, orange oil remained, and it was collected and stored for use in the experiment.

#### Specimen preparation

**Figure 1:**
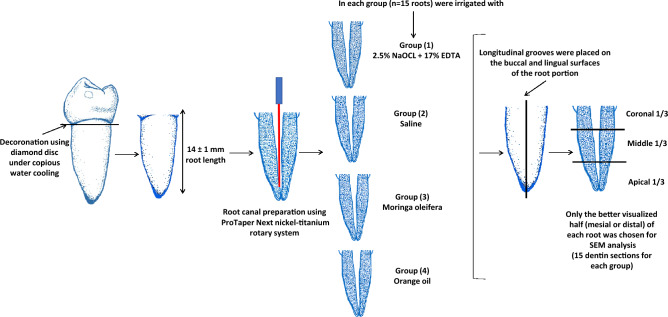
Schematic representation of specimen preparation.

All experiments were performed in accordance with relevant guidelines and regulations by the Research Ethics Committee (REC) of the Faculty of Dental Medicine for Girls, Al-Azhar University, Egypt (Approval No: REC-PD-24-01). Sixty human single-rooted mandibular premolars extracted for orthodontic purposes were selected for this study. Teeth with cracks, root caries, internal resorption or calcification were excluded. The teeth were radiographed to confirm the presence of a single canal. The selected teeth were cleaned thoroughly under running tap water using a soft toothbrush. Then, teeth were disinfected and stored at 4 °C in thymol solution until instrumentation. The teeth were de-coronated at cementoenamel junction using diamond discs (Diatech; Goltène; Switzerland) under copious water cooling. A uniform specimen length of 14 ± 1 mm was established for all teeth. Canal patency was checked using a # 10 K-file (K-file; MANI; Japan) until it became visible from the apex. After that, 1 mm was decreased from this length to determine the working length. The root apex of each tooth was sealed using sticky wax to avoid flowing of irrigants through the apex. The glide path was generated using a # 15 K file (K-file; MANI; Japan). Root canal preparation was performed using the ProTaper Next nickel-titanium rotary system (Dentsply, Maillfer, Switzerland). These movements were driven by an EndoEst motor (EndoEst motor; Geosoft Dent., Russia). They were used to size × 3 files for the crown-down technique, and speed and torque were adjusted according to the manufacturers’ instructions^2,21^. A schematic illustration of the specimen preparation is shown in Fig. 1.

#### Study design

In this study, the sample size of sixty human single-rooted mandibular premolar specimens was determined by dividing them into four equal groups, each comprising 15 specimens (n = 15). This allocation ensures a sufficient number of specimens for each irrigation protocol under investigation. The sample size of 15 per group was determined based on a 95% confidence interval and a power of 90% with an α error of 5%. The sample size was estimated using sample size calculator software program (G. power 3.19.2). The sixty human single-rooted mandibular premolars specimens were randomly divided into four equal groups according to one of the following irrigation protocols; **Group I:** 2.5% NaOCL (Clinix Endodontics, Dental Sky, UK) for 10 min followed by 17% EDTA for 10 min (Colgate Oral Care; Waverly; Australia.) (NE group). **Group II**: Root canals were irrigated with saline solution (sodium chloride 0.9%) (Baxter; Deerfield, Il; USA) (Saline group). In **Group III**: Root canals were irrigated with watery leaf extract of moringa oleifera (MO group). **Group IV**: Root canals were irrigated with orange oil (OO group). The irrigation protocol was based on previous studies showing that the cleansing efficient application time for smear layer removal was 10 min^35,36^. Prior to instrumentation, the root canal was irrigated with 2 mL of irrigating solution (NaOCL saline, moringa oleifera, orange oil) for 2 min. Irrigation with 2 ml of irrigating solution was done for 2 min between each file and after instrumentation, the canal was irrigated with 2 mL of irrigating solution for 2 min with a total volume of 10 mL in 10 min. In group I: 17% EDTA solution (5 mL) was utilized as a final irrigant for 10 min following saline separation. The irrigants were dispensed through a 31-gauge closed-end side-vented flexible stainless-steel irrigation needle (Navi-Tip, 31-ga side port; Ultradent, South Jordan, UT). The needle was inserted into the root canal 2 mm short of the working length without binding. Sterile paper points of size #30 (Dentsply, Maillefer, Ballaigues, Switzerland) were used to dry the canals after final rinsing with 5 ml of saline in all groups.

Longitudinal grooves were placed on the buccal and lingual surfaces of the roots without penetrating the root canal. The roots were separated into two halves with a chisel. For each root, the half containing the most visible part of the apex and total canal length was selected for better visualization of the irrigating solutions’ effect on the root canal walls (15 dentin sections for each group). Sequentially, they were cleaned with distilled water and a soft brush to remove dentin chips. A total of 60 root dentin sections of four groups were stored in well-sealed jars (jar for each group) filled with distilled water until further examination.

#### Scanning electron microscopy (SEM) assessment

All the teeth were sectioned along the long axis by a diamond disk alongside the root, thus creating a straight groove. Using a chisel, the splitting of teeth was carried out. For each root, the half containing the most visible part of the apex and the total canal length was chosen and kept in a 2% glutaraldehyde solution for 12 h; the other half was discarded. The selected sections were dried for 24 h in a desiccator under vacuum (Brown Vacuum Desiccator, China Supplier Chemistry Laboratory Glassware Clear, China) before SEM examination. The sections were mounted on an aluminum stub using carbon double sticky tape. They were then viewed using SEM (Prisma E SEM /FEI Quanta 3D 200i Edx/ Thermo Fisher Scientific—US) at an accelerating voltage of 20 kV. All images were captured at representative areas of the coronal, middle, and apical regions to evaluate the surface topography under 2000 × magnification^21^. After coding the specimens, SEM micrographs were observed by two expert evaluators who were ignorant of the coding of specimens. They scored the presence or absence of a smear layer in root canals using the scoring system of Torabinejad et al.^51^, and the results were analyzed descriptively.

#### Surface roughness measurements

Surface roughness analysis software (Scandium, Olympus, Adelaide, Australia) was used to obtain quantitative measurements of the three different regions (coronal, middle and apical) of each radicular dentin section of split root, from SEM images, using stereo pairs. A third party randomly selected the areas of the intraradicular dentin where the surface roughness was calculated. The roughness mean parameter of the absolute peak and valley heights (Ra) was selected to measure roughness from SEM images. The roughness of each sample was measured both in one dimension (along a line parallel as well as a line perpendicular to the radicular dentin region) and in two dimensions (along a surface)^37^.

#### Statistical analysis

The data were analyzed using IBM SPSS Statistics (Version 23). Surface roughness (Ra) data were explored for normality by checking data distribution and utilizing tests of normality (Kolmogorov–Smirnov and Shapiro–Wilk tests). Data exhibited a non-normal (non-parametric) distribution. Data were displayed as median, range, mean and standard deviation (SD) values. Kruskal–Wallis and Friedman tests were used to compare groups and root levels within each group, respectively. Also, Dunn’s test was used for pair-wise comparisons when both Kruskal–Wallis test and Friedman’s test were statistically significant at *p* value ≤ 0.05.

#### Ethics approval and consent to participate

All performed procedures of this study were carried out according to relevant ethical guidelines and regulations of Helsinki Declaration. The Research Ethics Committee (REC) of the Faculty of Dental Medicine for Girls, Al-Azhar University with the final code “REC-PD-24-01 on January 2024”, approved all experimental protocols (protocol code: P-PD-23-24). All methods and field studies on plants including the collection of plant material comply with relevant institutional, national, and international guidelines and legislation. For the collection of isolated teeth, informed consent was obtained from all participants.

## Results

**Figure 2:**
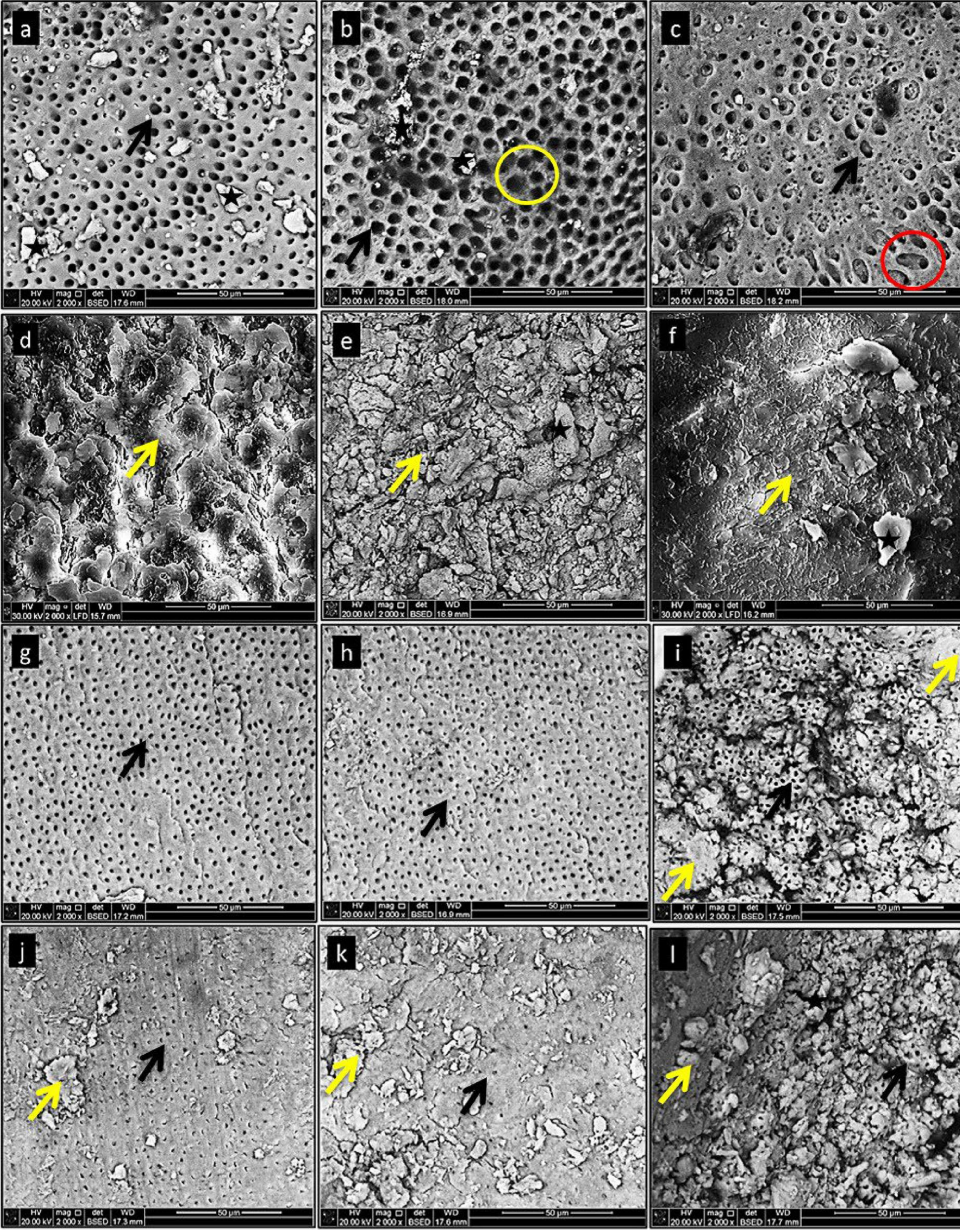
SEM images representing the IRD at different regions: (Fig. 2a, b, c), the NE group at the coronal (**a**), middle (**b**) and (**c**) apical regions shows opened dentinal tubules (black arrows) and dentin debris (stars). At (**b**) moderate erosion within intertubular dentin (yellow circle) and (**c**) severe erosion (red circle). (Fig. 2**d**, **e**, **f**), SEM images of IRD of saline group at coronal (**d**), middle (**e**), and apical sites (**f**) showing irregularities of smear layer (yellow arrow) and dentin debris (star). (Fig. 2**g**, **h**, **i**), SEM images of IRD of the MO group at coronal (**g**), middle (**h**) and apical (**i**) regions showing opened dentinal tubules (black arrow) and irregularities representing parts of smear layer in the apical part (yellow arrow). (Fig. 2**j**, **k**, **l**), SEM images of IRD of the OO group at the coronal (**j**), middle (**k**) and apical (**l**) sites show partially opened dentinal tubules (black arrow), irregularities of smear layer (yellow arrow). Original magnification 2000x.

The SEM images of coronal, middle, and apical regions of the IRD were evaluated for different irrigant groups (Fig. 2A-L). In the NE group (NaOCl 2.5% followed by EDTA 17%) (Fig. 2a,b,c) there was no smear layer, except for some dentin debris, which was scattered in the coronal, middle and apical parts. Dentinal tubules were opened and widened and exhibited variable shapes. Moderate erosion was observed in the intertubular dentin, especially in the middle third (Fig. 2b). However, some dentinal tubules exhibited severe erosion through their connection, as shown in the apical third (Fig. 2c). In the saline group (Fig. 2d,e and f), heavy surface coverage of smear layer was revealed with a smear layer with no opened tubules through IRD. Furthermore, dentin debris was shown through IRD of the apical third region (Fig. 2f). For moringa group (MO) (Fig. 2g,h, and i), there was no visible sign of erosion, and the dentinal tubules were opened through different regions. The diameter of tubules also had uniform outlines with completely cleaned without debris at the coronal and middle regions of IRD (Fig. 2g,h). However, some of the tubules were plugged and some irregularities representing the smear layer remained in the apical region (Fig. 2i). In the orange peel oil group (OO), opened dentinal tubules were observed to be narrower and lesser in extent (Fig. 2j,k,l) than the corresponding tubules in the NE (Fig. 2a-c) and MO groups (Fig. 2g-i). Partially opened dentinal tubules were more prevalent in the coronal region (Fig. 2j) than in the middle and apical regions (Fig. 2k, l). There were many irregularities on the IRD surface as remnants of the smear layer.

**Figure 3:**
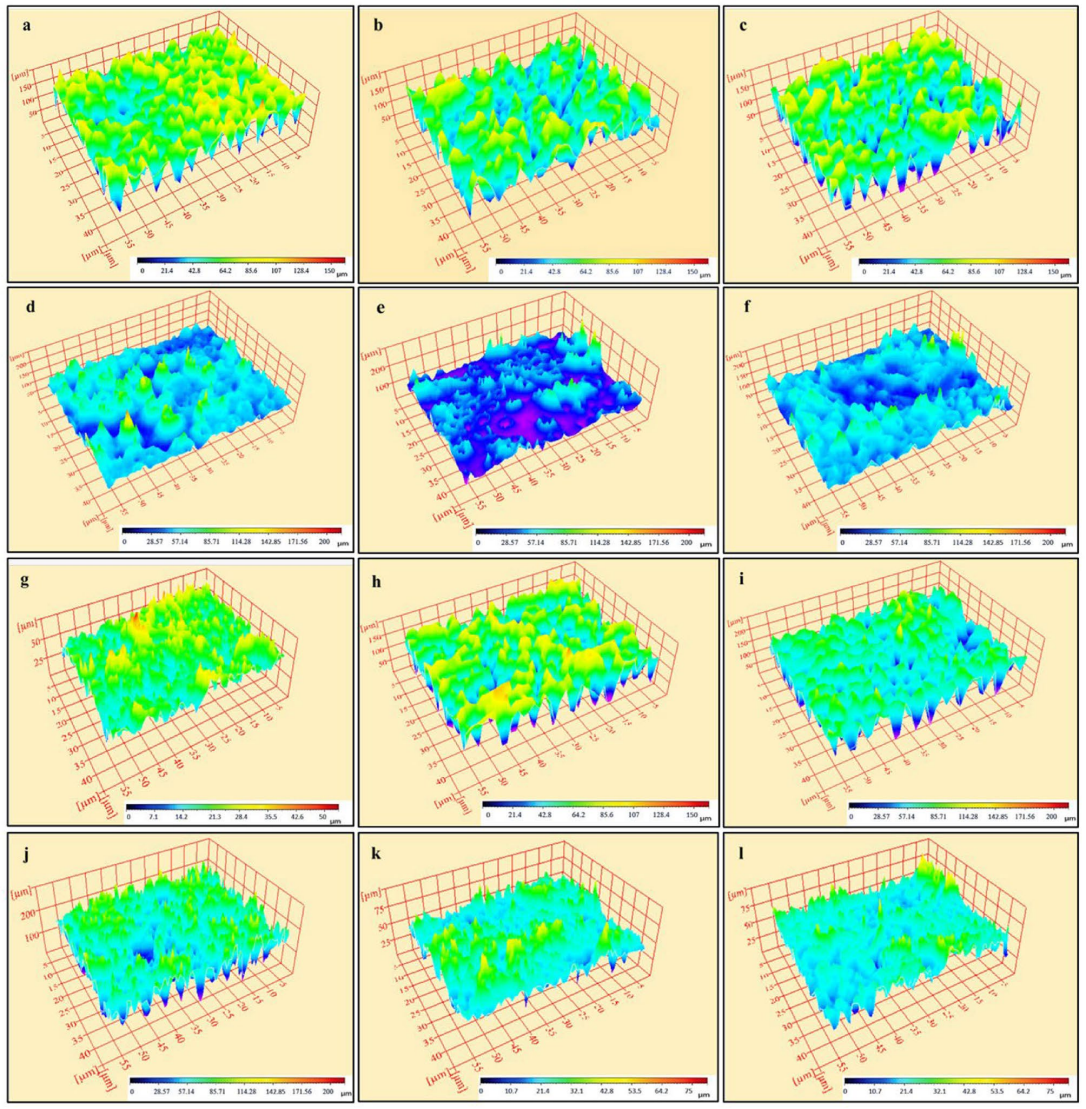
(**a**-**l**) 3D images of the surface topography of different IRD regions following the irrigation protocols. **a**, **b**, **c**: Coronal, middle and apical parts of IRD in NE group. **d**, **e**, **f**: Coronal, middle and apical parts of IRD in saline group. g, h, i: Coronal, middle and apical parts of IRD in the MO group. **j**, **k**, **l**: Coronal, middle and apical parts of IRD in OO group. The color indicates the measured height in micrometers (µm) above the lowest point for each surface.

Surface roughness analysis was carried out to obtain quantitative results representing in 3D images of the surface topography of different regions of IRD following the irrigation protocols in Fig. 3a-l. These images reveal that each sample has a different morphology, and the surface for the group NE and MO at the coronal and middle have almost the highest roughness with a minimum surface roughness at the apical third. Moreover, the presence of either a heavy smear layer in saline group or partial coverage as in OO group could decrease roughness. These 3D images are demonstrated by the overall effect of the measured surface roughness parameter (R_a_). Regarding Table 1. the median, range, mean and standard deviation (SD) of roughness (R_a_) values in micrometers (µm) for the different irrigant groups and regions of IRD were presented. There was a statistically significant difference in the roughness mean parameter (R_a_) values in coronal, middle and apical regions of IRD between groups (*p*-value = 0.007) (Table 1) which confirmed. A pairwise comparison indicated that there was a statistically significant difference between them. The NE group had the highest R_a_ value, followed by the MO and OO groups, while the saline group had the lowest value. However, there was no statistically significant difference between saline and orange oil groups in the middle and apical parts, as both had the lowest R_a_ with no statistically significant difference. For the roughness mean parameter (R_a_) within each group, there was no statistically significant difference between R_a_ values between coronal, middle and apical regions *p*-value = 0.091, 0.819, 0.819 and 0.549 respectively.

**Table 1:** Comparison between the roughness mean parameter (R_a_) (µm) in different groups and comparing IRD levels within each group. The different letters indicate significant difference at *p* ≤ 0.05.

Intra radicular dentin level	NE		Saline		MO		OO		*p*-value	Effect size (Eta squared)
	Median (Range)	Mean (SD)	Median (Range)	Mean (SD)	Median (Range)	Mean (SD)	Median (Range)	Mean (SD)		
Coronal	20.4 (16.2–30.2) ^A^	22.6 (5.4)	7.1 (5–10) ^D^	7.3 (1.8)	13.7 (5.8–19.2) ^B^	12.2 (5.6)	9.5 (8.1–16.2) ^C^	10.5 (3.4)	0.007	0.686
Middle	21.8 (13.8–26.1) ^A^	20.9 (5.4)	8.1 (4.1–9.6) ^C^	7.6 (2.1)	12.5 (6.1–18.8) ^B^	13.1 (5.2)	7.4 (6.6–9.1) ^C^	7.8 (1.1)	0.009	0.703
Apical	13.3 (10–24.8) ^A^	15.2 (5.6)	9.1 (6.5–12.1) ^C^	9.1 (2.4)	10.9 (7.3–15.1) ^B^	10.9 (3.3)	9 (6.6–10.2) ^C^	8.5 (1.4)	0.046	0.406
Overall	17.6 (15.8–23.8) ^A^	19.5 (3.6)	7.4 (7.2–9.6) ^C^	8 (1.1)	12.8 (7.1–17) ^B^	12 (4)	8.7 (8.2–10.1) ^C^	8.9 (0.7)	0.007	0.771
*p*-value	0.091		0.819		0.819		0.549			
Effect size (w)	0.480		0.040		0.040		0.120			

## Discussion

An advanced technology based on scale-sensitive fractal analysis of high-resolution and three-dimensional surface reconstructions was used in the current study to evaluate dentin surface roughness following different irrigation protocols^29^. This study is the first to provide a detailed analysis of biological effect of herbal irrigants on the surface texture of intraradicular dentin, which is very important in ensuring the adaptation of the root canal filling materials through micromechanical connection to the canal dentin. One of the most common techniques for assessing the effect of irrigation on intracanal dentin tissue (IDR) is surface roughness^38^. The surface roughness R_a_ parameter values of irrigant groups were statistically significantly different (*p*-value ˂0.007) through all IRD levels (coronal, middle, and apical parts). The NE group exhibited the most significant changes in R_a_. Then, the MO had a significantly lower R_a_ than the NE group, followed by the OO group; hence, the null hypothesis of this research was rejected. However, there was no statistically significant difference in the R_a_ values between the OO and saline groups as well as at different IRD levels of MO and OO groups. So, the second and third hypotheses of this study was not rejected.

Chemical irrigants may alter the chemical and physical properties of root dentin^39^. However, the use of these chemicals might pose toxic hazards to periapical tissues^12,40^. Additionally, concerns about the effect of certain chemicals on dentin erosion persist^4^. Various studies have shown that the chelating action of EDTA causes dentin erosion due to hyperactivity^2,41^. However, when NaOCl is used after EDTA, the collagen that has already been exposed to demineralizing agents is subjected to the dissolving effect of NaOCl^42^. Hence, in the present study, it is suggested that NaOCl be used prior to EDTA, as the hydroxyapatite coating appears to shield the collagen fibers from the dissolving effect of NaOCl^2^. Moreover, a combination of NaOCl and EDTA (NE) was used sequentially in the present study as the gold standard for producing rough surfaces and effectively removing smear layer components. This was because there is still no irrigant in the dental market capable of achieving complete debridement^43^.

In fact, these chemicals decrease the hardness of dentin and negatively affect the adhesion of sealers to intraradicular dentin (IRD)^18^. Accordingly, research has focused on creating compatible irrigant solutions from medicinal herbs. Moringa oleifera (MO) is a promising medicinal herb that protects enamel and dentin, preventing erosion^12^. Furthermore, orange oil (OO) is an essential oil that is recognized as biocompatible and has an impact on smear layer removal^22^. MO and OO irrigants were used in the present study for a period of 10 min. This was due to the simulation of the clinical situation of the NaOCl and EDTA protocols during irrigation, and depending on the results of previous studies showing that the most efficient application time for smear layer removal was 10 min^35,36^. Saline was used as a negative control because it has no dissolving capacity or effect on dentin due to its near-neutral pH^44^. While the positive control of NaOCl and EDTA was used to compare the effects of MO and OO irrigants on the surface texture of IRD to their known effects from previous literature^2^. Currently, there is no available data on the effect of the surface roughness of herbal MO and OO on IRD following irrigation.

Although there are common methods for evaluating surface roughness, they allow only the investigation of relatively small surface areas and may not provide enough data regarding surface features^1,45^. In addition, the most widely used reported roughness measurement method is the average roughness parameter (R_a_)^38^. Standardized parameters from engineering applications are employed in 3D quantitative analysis, allowing reliable characterization on a micrometre scale^1^. In addition, analysis of SEM imaging of IRD regions allowed for qualitative biological comparisons after irrigation^46^.

The efficacy of eliminating the smear layer was demonstrated using 2.5% NaOCl plus 17% EDTA (NE group). The dentinal tubules were opened and widened through IRD regions. However, such action was accompanied by moderate to severe dentin erosion. The SEM results were consistent with those of the changes in the NE surface roughness. Among all the groups, the NE combination had the highest IRD surface roughness (Ra), which was statistically significant. It was reported that using NaOCl plus EDTA increases the roughness of the dentin wall of the root canal^2^. EDTA acts through chelation and protonation mechanisms since hydrogen ions in the carboxylic group of EDTA replace calcium ions in hydroxyapatite^39^. As previously mentioned, NaOCl dissolves and removes the dentin collagen matrix, contributing to dentin surface alterations and could improve the ability of the chelating agent to penetrate and influence dentin^2,38^. Furthermore, the action of chelation agents is intense in the underlying substrate after smear layer removal^47^. Thus, the combined effect of both materials may be the reason for the increased erosion and surface roughness changes in IRD. These findings are consistent with previous studies that used 17% EDTA to effectively eliminate the smear layer. EDTA increased, the surface roughness of IRD when applied as a final irrigant after NaOCl^48,49^.

Based on the present SEM analysis, MO was found to be an effective irrigant for removing the smear layer and opening dentinal tubules without causing any erosion. Additionally, it noticeably decreased the deposits in the coronal, middle, and apical parts to a small amount. There was considerable IRD surface roughness in the MO group compared to the NE combination and those of the OO group. The presence of flavonoids and phenolic acids in MO might contribute to the preservation of dentinal tubules through an increase in intertubular dentin and proper Ra values^19^. Flavonoids and phenolic acid cause demineralization of the inorganic part of the smear layer, liberating calcium ions (Ca^2+^) and hydrogen phosphate (HPO_4_^2-^) that are soluble in water^50^.These findings agreed with recent studies revealing that moringa extract resulted in effective smear layer removal and widening of dentinal tubules^14,18^. In a study conducted by Natsir et al., demonstrated that moringa leaf extract had a favourable effect on removing the smear layer^19^.

Concerning the OO irrigant, fewer quantities of smear layer were removed, and dentinal tubules were partially opened in the coronal region. Additionally, through IRD regions, the Ra values of the OO irrigant were substantially lower than those of the MO and NE irrigants. The cleaning action of d-limonene, flavonoids, and citric acid in OO irrigant could be responsible for the partial smear layer elimination in the coronal region and a certain decrease in dentin roughness^20,21^. Despite the presence of citric acid in OO, Torabinejad et al. reported that citric acid-containing irrigants had no significant effect on dentin structure when used alone^51^. The lesser efficacy of OO irrigant than that of NE or MO could be due to the deficiency of essential acid metabolites for dissolving the smear layer. Nevertheless, the mild effect of OO decreased significantly in the middle and apical IRD regions. This difference might be attributed to the greater surface tension and viscosity of the oily nature of the orange extract than those of the NE and MO irrigants. This rheological nature results in reduced wettability, preventing orange oil from penetrating down into the root canal^21^.

These results agreed with those of the previous study, which stated that orange oil extract irrigant could be able to partially dissolve the smear layer from the apical part^21^. On the other hand, the current results contrast with a previous study. This could be caused by differences in irrigation techniques and timing.

Furthermore, our findings indicated that, compared to the OO irrigant, MO had significantly greater Ra values. This difference might be attributed to the lower viscosity and surface tension of the watery extract of moringa. Fluid surface tension and viscosity largely influence the ability of an irrigant to penetrate dentin and its ability to spread on dentin surfaces^52^.

Saline, as a negative control, caused the least amount of smear layer elimination. A homogeneous smear layer was detected, with no appearance of opened dentinal tubules. The resultant surface roughness of herbal irrigants, except for the middle and apical parts irrigated with OO, was significantly greater than the roughness produced by saline. This could be due to its near-neutral pH, which might not cause any changes in the inorganic or organic components of dentin^44^.

This study revealed that there was no statistically significant difference in surface roughness at different root levels within each group. This was in full agreement with the findings of a previous study showing no statistically significant difference in smear layer elimination between the apical, middle, and coronal thirds^21^.

The current in vitro results obtained using Moringa oleifera (MO) as an endodontic irrigant were promising. Proper surface roughness without erosion through the intraradicular dentin regions was achieved compared to that occurring with the NaOCl plus EDTA irrigants (NE). However, the present study was performed under in vitro conditions, and although every effort was made to simulate a clinical situation, the oral cavity is a complex structure, and an exact simulation cannot be reproduced.

Further research is needed to reveal how dentin hardness and biomechanical properties are affected by these herbal irrigants. Also, more research is required to evaluate how the roughness obtained by the aqueous Moringa solution might affect the bond strength of different root canal fillings. Additional studies should evaluate smear layer removal using micro-CT.

## Conclusions

Under the limitations of the current study, a new methodology of 3D dentin surface roughness evaluation following irrigation using herbal Moringa oleifera as an irrigant might be advantageous. It has a favourable effect on dentin microstructures. Considerable roughness and smear layer removal could make Moringa extract a substitute for the NaOCl plus EDTA regimen. Further study is required to evaluate the bond strength of the endodontic sealer to the root canal wall after irrigation with this experimental herbal irrigant.

## Data Availability

The datasets used and/or analysed during the current study available from the corresponding author on reasonable request.
